# Interim Acrylic Plug-Type Obturator Prosthesis Following Marsupialization of a Maxillary Dentigerous Cyst in an Adolescent: A Case Report

**DOI:** 10.7759/cureus.93603

**Published:** 2025-09-30

**Authors:** Chandrama Pratap, Rishabh Khare, Chingkheinganbi Nameirakpam, Sarjubala Nongthombam

**Affiliations:** 1 Prosthodontics, Swami Vivekanand Subharti University, Meerut, IND; 2 Prosthodontics, JN Kapoor DAV Dental College, Yamunanagar, IND

**Keywords:** acrylic plug prosthesis, interim obturator, left maxilla, marsupialization, dentigerous cyst

## Abstract

This case report details the interdisciplinary management of a large dentigerous cyst located in the left maxillary region of a 16-year-old male patient. Marsupialization performed by the Oral and Maxillofacial Surgery Department created a surgical defect, which was successfully rehabilitated using an acrylic plug-type interim obturator fabricated by the Department of Prosthodontics. Sequential clinical and laboratory images demonstrate the treatment phases. This case underscores the importance of surgical-prosthodontic collaboration in restoring function, esthetics, and hygiene in maxillofacial cystic lesions.

## Introduction

Dentigerous cysts are the second most common odontogenic cyst, frequently encountered in adolescents and typically associated with the crown of impacted or unerupted teeth. Large cystic lesions in the maxillary region can cause swelling, displacement of teeth, and maxillary bone expansion, leading to esthetic and functional disturbances. Conservative management via marsupialization reduces surgical morbidity by decompressing the cyst and preserving vital anatomy but results in an intraoral defect that requires interim management to maintain oral function and hygiene. Acrylic plug-type obturators are widely used interim prostheses designed to seal surgical defects, facilitating patient comfort and cavity healing during this phase [1].

## Case presentation

Patient profile and presentation

A 16-year-old male patient was referred with a chief complaint of a painless swelling in the anterior region of the left maxilla. Clinical examination revealed mild facial asymmetry with a firm swelling over the left upper lip area. Intraorally, buccal cortical plate expansion was noted in the left maxillary canine region, with overlying mucosa intact. The patient had no relevant medical history.

Pathological Findings and Preoperative Diagnosis

The procedure was performed in the OMR Department. Fine needle aspiration cytology (FNAC) was conducted first, revealing pathological findings indicative of a benign cystic lesion with clusters of epithelial cells and a mucoid fluid background. Following the FNAC results, marsupialization was carried out as a conservative surgical treatment to decompress the cystic lesion. During the procedure, the cyst wall was incised and sutured to form a permanent open pouch, allowing continuous drainage and preventing fluid accumulation. The pathological findings from the FNAC guided the surgical approach and helped in confirming the diagnosis before marsupialization. The procedure and postoperative management were conducted under standard protocols in the department.

Radiographic findings

**Figure 1:**
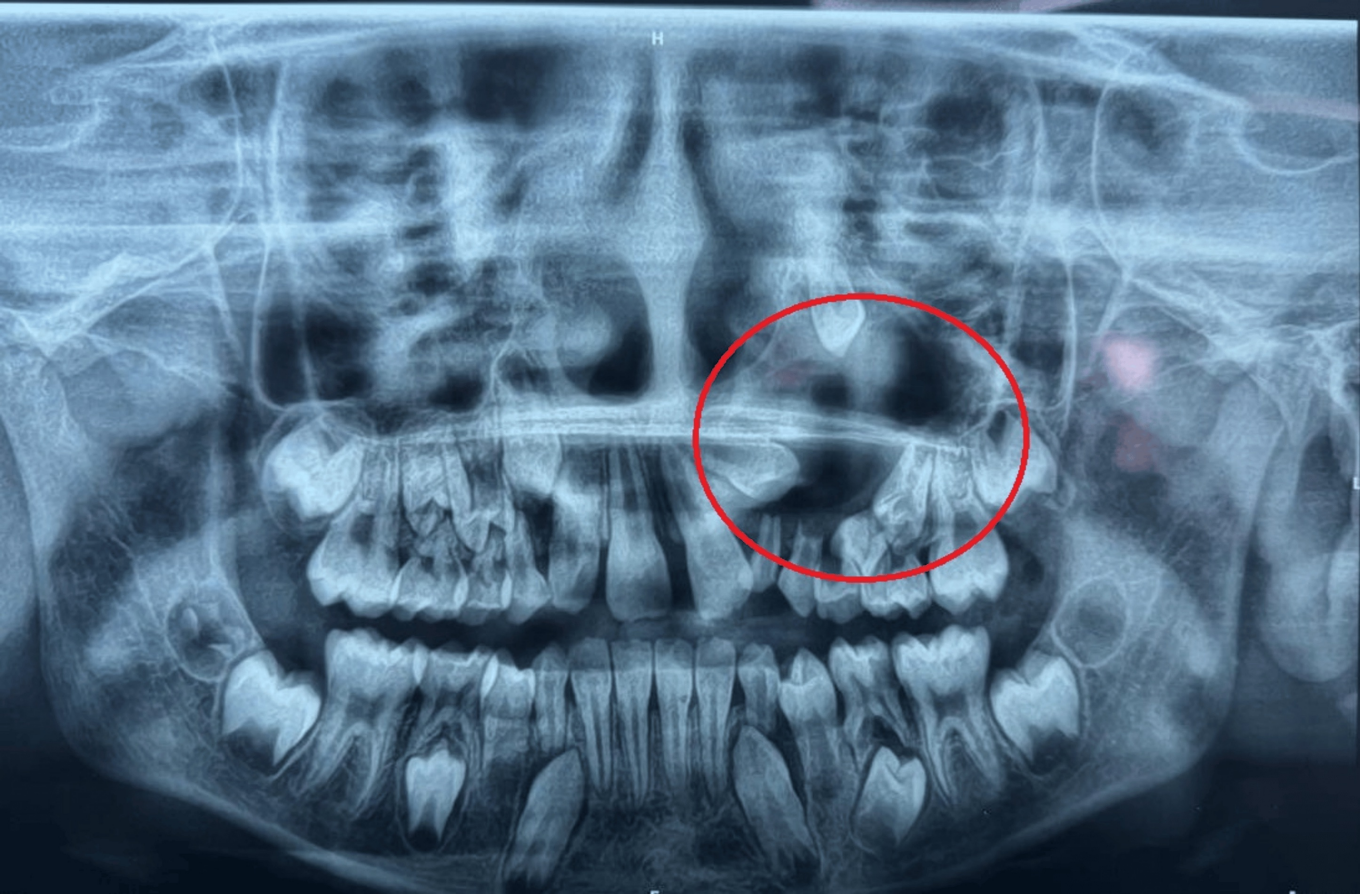
Orthopantomogram (OPG) showing well-defined unilocular radiolucency in the left maxillary region A well-defined unilocular radiolucency in the left maxillary region, associated with an unerupted maxillary canine. The lesion shows corticated borders, displacement of adjacent teeth, and extension into the floor of the maxillary sinus, consistent with the features of a dentigerous cyst in the upper left tooth region.

A panoramic radiograph revealed a well-circumscribed unilocular radiolucency enveloping the crown of the impacted left permanent maxillary canine, extending superiorly towards the maxillary sinus floor and causing cortical expansion (Figure 1). The lesion was diagnosed radiographically as a dentigerous cyst localized specifically in the left anterior maxilla, with a size of 2.5 cm × 2.8 cm.

Surgical management

**Figure 2:**
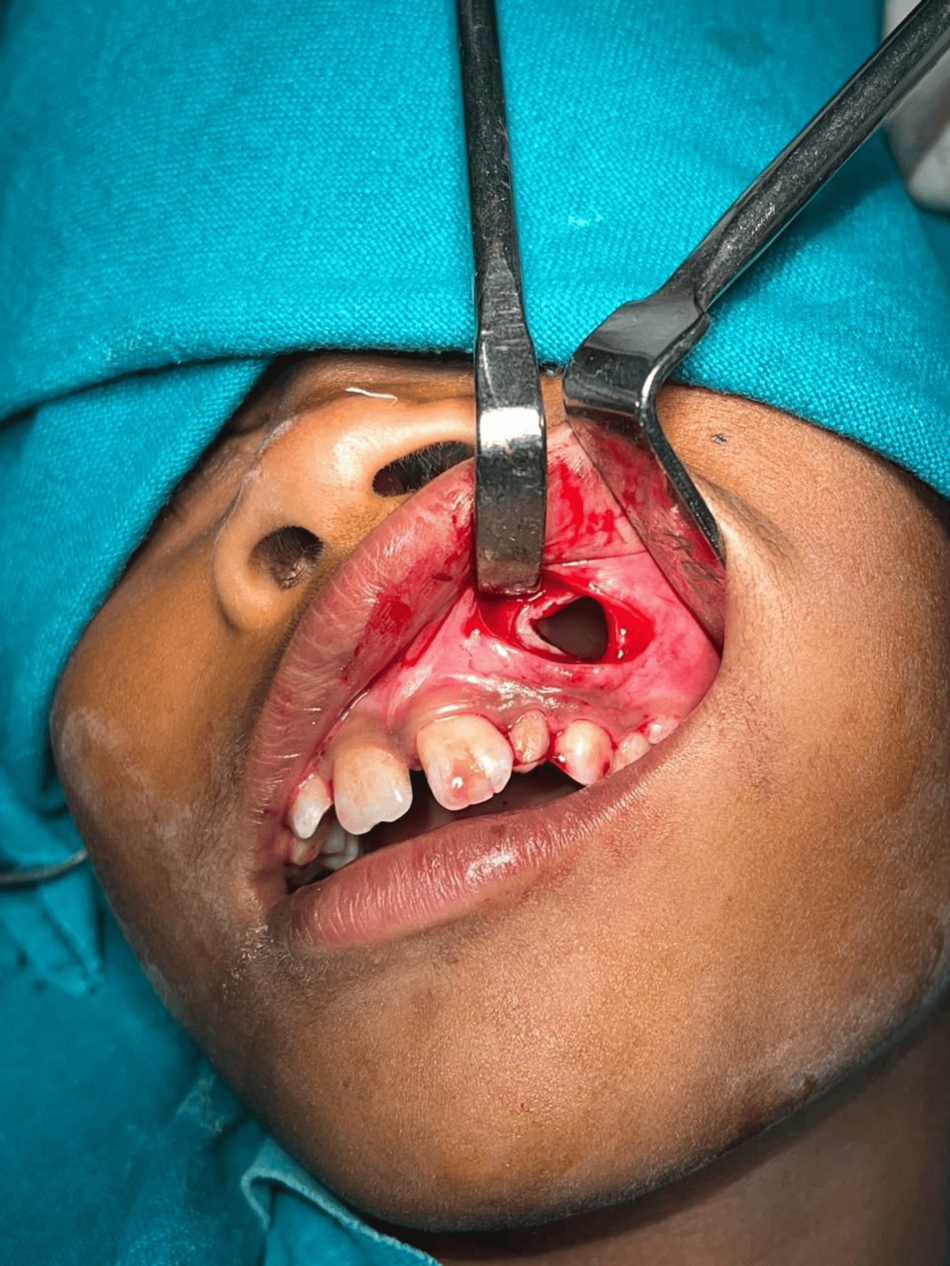
Intraoperative view: marsupialization defect in the anterior maxilla

The patient was scheduled for marsupialization under local anesthesia in the Oral and Maxillofacial Surgery Department. A mucosal window was created in the buccal vestibule overlying the lesion. The cystic lining was carefully incised and sutured to the oral mucosa, creating a permanent opening to decompress the cyst (Figure 2). The marsupialization aimed at gradual cyst volume reduction while preserving adjacent anatomical structures [1].

Prosthodontic rehabilitation

Impression and Framework Design

**Figure 3:**
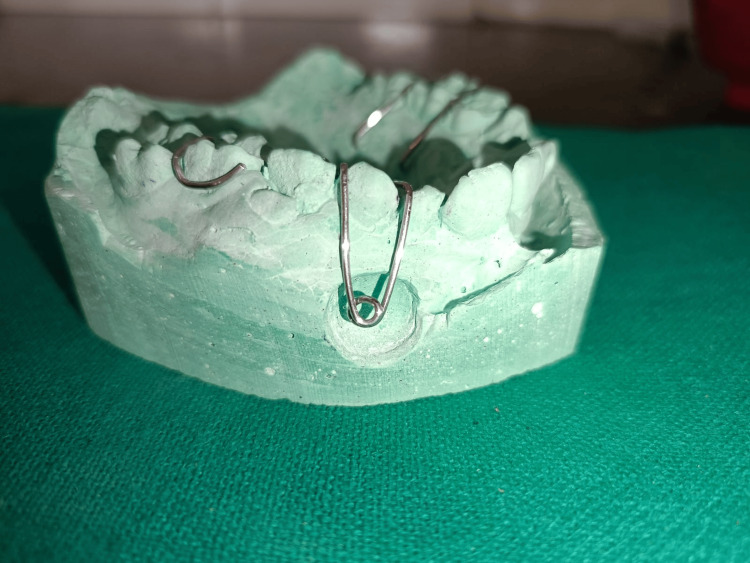
Lab cast with a wire framework for obturator fabrication Laboratory cast displaying the wire framework fabricated for the obturator.

Following adequate surgical wound dressing and initial healing, an impression was made of the maxillary arch, capturing the defect morphology accurately. A detailed diagnostic cast was prepared on which a retentive wire framework was constructed. Clasp arms engaged the adjacent stable teeth, while a rigid loop projected over the defect site for prosthesis support (Figure 3) [2]. 

Fabrication of the Acrylic Plug-Type Obturator

**Figure 4:**
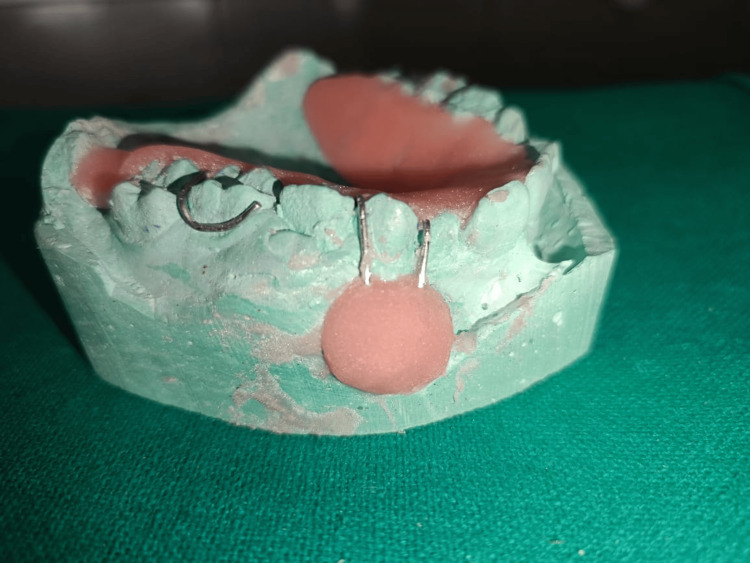
Acrylic plug-type obturator on the cast Acrylic plug-type obturator fabricated on the cast before insertion.

The defect area was blocked out on the cast to prevent acrylic flow beyond the desired limits. Heat-polymerized acrylic resin was processed to form a bulbous plug conforming to the cyst cavity dimension, ensuring firm but comfortable retention. The prosthesis sealed the surgical opening to prevent contamination and improved functional contours (Figure 4) [3].

Delivery and Follow-Up

**Figure 5:**
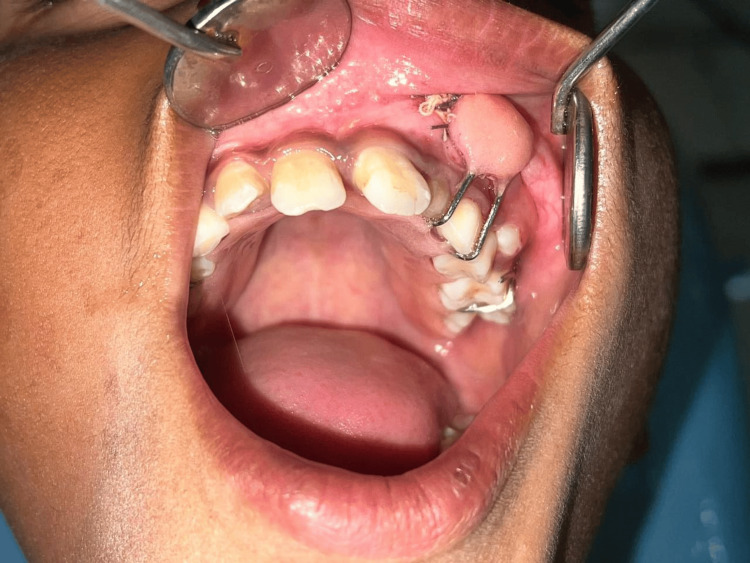
Immediate intraoral placement of the interim obturator Immediate intraoral placement of the interim obturator prosthesis.

The interim obturator was inserted intraorally immediately post-fabrication. It snugly filled the defect, was retained by the wire clasps, and allowed unhindered oral functions such as speech and mastication (Figure 5) [4]. The patient was counseled on prosthesis removal for hygiene maintenance and given instructions on cavity irrigation and dietary modifications. Follow-up visits over three weeks showed progressive adaptation, improved phonation and mastication, and favorable wound healing with reduction in cavity size [1].

## Discussion

Marsupialization remains a conservative yet effective choice for managing large cystic lesions, especially in young patients. The creation of a surgical opening necessitates an interim prosthetic solution to maintain cavity patency and prevent infection. Acrylic plug-type obturators meet these criteria efficiently due to their adjustability, ease of fabrication, and patient comfort.

Management of a cyst in the left maxillary anterior region poses unique challenges due to esthetic considerations and structural proximity to the maxillary sinus and nasal cavity. Early prosthetic intervention with obturators prevents food accumulation, aids speech, and stabilizes the surgical site, accelerating recovery.

The literature supports the indispensable role of such prostheses in transitional care post marsupialization as part of a multidisciplinary treatment plan. Successful outcomes rely on careful prosthesis design, patient compliance, and regular monitoring [3-5].

## Conclusions

This case demonstrates that marsupialization, followed by interim prosthetic obturation using an acrylic plug, is a viable approach to manage large dentigerous cysts in the left maxilla of adolescents. The combination of surgical and prosthodontic care restores oral function, maintains hygiene, and supports psychosocial well-being, exemplifying effective interdisciplinary maxillofacial management.
